# 

**Published:** 1858-03

**Authors:** 


					﻿No. X.]
MARCH.
[Vol. XTII.
BUFFALO
MEDICAL JOURNAL
AND
Meview
or
MEDICAL AND SURGICAL SCIENCE.
SANFORD B. HUNT, M. D.,
EDITO El.
AUSTIN FLINT, Jr., M. D.,
ASSOCIATE EDITOR.
- BUFFALO, N. Y.:
E. R. JEWETT <fc CO.’S STEAM I' R ES 8.
Commercial Advertiser Buildings.
185S.
Price $2.00 in advance, or $2.50 at the end of three months.
				

